# Transcriptomics and Metabolomics Analysis Provides Insight into Leaf Color and Photosynthesis Variation of the Yellow-Green Leaf Mutant of Hami Melon (*Cucumis melo* L.)

**DOI:** 10.3390/plants12081623

**Published:** 2023-04-12

**Authors:** Hongwei Han, Yuan Zhou, Huifang Liu, Xianjun Chen, Qiang Wang, Hongmei Zhuang, Xiaoxia Sun, Qihua Ling, Huijun Zhang, Baike Wang, Juan Wang, Yaping Tang, Hao Wang, Huiying Liu

**Affiliations:** 1 Key Laboratory of Special Fruits and Vegetables Cultivation Physiology and Germplasm Resources Utilization of Xinjiang Production and Construction Corps, Department of Horticulture, College of Agriculture, Shihezi University, Shihezi 832003, China; hhwei2021@xaas.ac.cn (H.H.);; 2 Key Laboratory of Horticulture Crop Genomics and Genetic Improvement in Xinjiang, Institute of Horticultural Crops, Xinjiang Academy of Agricultural Sciences, Urumqi 830002, China; 3 National Key Laboratory of Plant Molecular Genetics, CAS Center for Excellence in Molecular Plant Sciences, Institute of Plant Physiology and Ecology, Chinese Academy of Sciences, Shanghai 200030, China; 4 School of Life Science, Huaibei Normal University, Huaibei 235000, China

**Keywords:** Hami melon mutant, chloroplast, photosynthesis, photosynthetic carbon assimilation, ROS, transcriptome, metabolism

## Abstract

Leaf color mutants are ideal materials for studying the regulatory mechanism of chloroplast development and photosynthesis. We isolated a cucumis melo spontaneous mutant (MT), which showed yellow-green leaf phenotype in the whole growing period and could be inherited stably. We compared its leaves with the wild type (WT) in terms of cytology, physiology, transcriptome and metabolism. The results showed that the thylakoid grana lamellae of MT were loosely arranged and fewer in number than WT. Physiological experiments also showed that MT had less chlorophyll content and more accumulation of reactive oxygen species (ROS) than WT. Furthermore, the activity of several key enzymes in C_4_ photosynthetic carbon assimilation pathway was more enhanced in MT than WT. Transcriptomic and metabolomic analyses showed that differential expression genes and differentially accumulated metabolites in MT were mainly co-enriched in the pathways related to photosystem-antenna proteins, central carbon metabolism, glutathione metabolism, phenylpropanoid biosynthesis and flavonoid metabolism. We also analyzed several key proteins in photosynthesis and chloroplast transport by Western blot. In summary, the results may provide a new insight into the understanding of how plants respond to the impaired photosynthesis by regulating chloroplast development and photosynthetic carbon assimilation pathways.

## 1. Introduction

The leaf color of higher plants is attributed to various leaf pigments, mainly chlorophyll, carotenoids and anthocyanin. Photosynthetic organisms exhibit a green color due to the accumulation of chlorophyll pigments in chloroplasts [1]. Chlorophyll, the most abundant pigment on earth, is necessary for photosynthesis for light harvesting and driving electron transport in the reaction centers in higher plants [2]. The changes of leaf color, usually caused by abnormal chlorophyll metabolism, significantly affect photosynthesis efficiency and then influence the growth and development of plants [3]. Therefore, leaf color mutants are ideal materials for studying chloroplasts development and the regulation mechanism of photosynthesis [4]. Different leaf color phenotypes include albino, faded green, light green, holding green, striped and zebra, albino re-green, dark green, and purple [5], and are widely found in higher plants. In recent years, more and more leaf color mutants have been identified and used to reveal the mechanisms for the regulation of chloroplast development and function, chlorophyll biosynthesis, and photosynthesis [6,7,8,9] in various plant species such as rice [6,10,11], wheat [12,13], maize [14,15], Arabidopsis [16,17], kale-type oilseed rape [18], Camellia sinensis [7,19], melon [20], and other cereals [21].

Because a series of enzymatic steps are involved in chlorophyll biosynthesis, blocking any step of chlorophyll synthesis in plants causes low chlorophyll content, resulting in green-deficient leaf color [22,23]. Accordingly, studies of leaf color variation have generally focused on chlorophyll biosynthesis and degradation. For example, a large number of rice leaf color varieties with chlorophyll deficiency were identified to be related to the gene mutations of chlorophyll synthesis and metabolism pathways of magnesium chelatase [1,24], OsGluRS encoding a putative glutamyl-tRNA synthetase [25], OsCAO1 encoding the chlorophyll a oxidase [26,27], DVR encoding a covalent reductase [28,29], and POR encoding the protochlorophyllide oxidoreductase [8]. The synthesis of 5-aminolevulinic acid (ALA) is the rate-limiting step for the formation of all plant tetrapyrroles, including chlorophyll and heme. Glutamyl-tRNA reductase is the first committed enzyme of this pathway and is encoded by a small family of nuclear HEMA genes, in which the mutations of HEMA1, HEMA2, and HEMA3 were also identified to be related to leaf color variation [30].

Nuclear and chloroplast genes act in a coordinated manner to regulate chloroplast development involving chloroplast and nuclear gene transcription, protein translation, processing modification, protein folding and degradation, thylakoid formation, and pigment synthesis [21,31,32]. Chloroplasts consist of a complex thylakoid membrane system that embeds pigment-protein supercomplexes, including photosystem I (PSⅠ), photosystem Ⅱ (PSⅡ), cytochrome b6f, and ATP-synthase [33]. Mutations or down-regulation of the genes encoding PSⅡ light-harvesting antenna protein complexes (LHCⅡ), which are responsible for capturing and transmitting light energy to the reaction centre of the thylakoid membrane, were reported to cause chlorophyll deficiency in Arabidopsis and Lagerstroemia indica [34]. Mutations in genes encoding signal recognition granule proteins SRP43 and SRP54 [35,36,37] and ABC transporter proteins [38] were also considered to be related to leaf color variations in rice and Arabidopsis, respectively. RNA polymerase (PEP) plays a vital role in the transcription of plastid tRNAs and photosynthetic machinery. The deletion of PEP-related proteins ZmPTAC2, ZmMurE, ZmPTAC10, ZmPTAC12, or ZmPRIN2 causes a yellow leaf phenotype, and the mutant cells contain fewer plastid ribosomes and photosynthetic complexes [39]. RNA editing is a post-transcriptional modification process that changes the sequence of RNA molecules so that the information in the mature RNA differs from that defined in the genome [40], plays a key role in organelle gene expression. Several organelle RNA recognition motif-containing (ORRM) proteins are known to be essential RNA editing factors. ORRM1 controls 62% of chloroplast editing sites in Arabidopsis and 81% of editing sites in maize, with the Zm-orrm1 mutant exhibiting a pale green phenotype [41]. A genetic screen in yellow-leaf soybean detected a point mutation in YL (YELLOW LEAF), a chloroplast-localized gene homologous to the Arabidopsis ORRM1 (ORGANELLE RRM PROTEIN 1), which regulates chloroplast RNA-editing and photosynthesis [42]. In addition, abnormalities in the metabolism of other substances such as carotenoids and sugars can also regulate chloroplast development through the plastid-to-nucleus retrograde signaling pathway [43,44].

Hami melon is an important cultivation type of melon (*Cucumis melo* L.), which is one of the most important fruiting vegetables in the worldwide [45]. There has been limited research conducted on the mechanism of leaf color mutation in melon due to its narrow genetic background and the low frequency of natural mutations. Shao Qin (2013) performed proteomic analysis using 2-DE and GC-MS; the result showed that the contents of glutamate (Glu) and aspartic acid (Asp) were significantly down-regulated in the leaf yellowing mutant of a thin-skinned melon (9338-1) [46]. Early termination of protein translation caused by a CmGLK transcription factor mutation induced the yellow-green leaf phenotype in the thin-skinned melon mutant (M68) [20,47]. However, studies of leaf color mutants in Hami melons have not been reported. Recently, we isolated a natural chlorophyll-less mutant (MT) from the local variety ‘paotaihong’ of Hami melon in Xinjiang. Interestingly, we previously showed that the photosynthetic efficiency of MT was not compromised, and the activity of key enzymes of carbon assimilation was significantly higher than that of the wild type, such as ribulose bisphosphonate carboxygenase (Rubisco), pyruvate phosphate dikinase (PPDK) and phosphoenolpyruvate carboxylase (PEPC) [48], indicated that the mutation may induce a complex adaptive regulation.

In this study, we compared the chloroplast ultrastructure, pigment content, ROS and enzyme activity of the antioxidant system between the leaves of MT and the wild type. We also analyzed differential changes in gene expression and metabolite accumulation by transcriptome sequencing and tandem mass spectrometry (LC-MS/MS). Additionally, we detected the abundances of four proteins related to chloroplast development and photosynthesis by Western blot. This analysis of the differentially accumulated metabolites (DAMs) and differential expression genes (DEGs) between mutant and wild-type leaves could provide novel insights into the potential molecular mechanism underlying the MT phenotype and provide a useful reference for plant leaf coloration studies.

## 2. Results

### 2.1. Agronomic Traits and Inheritance of the Mutant

**Figure 1:**
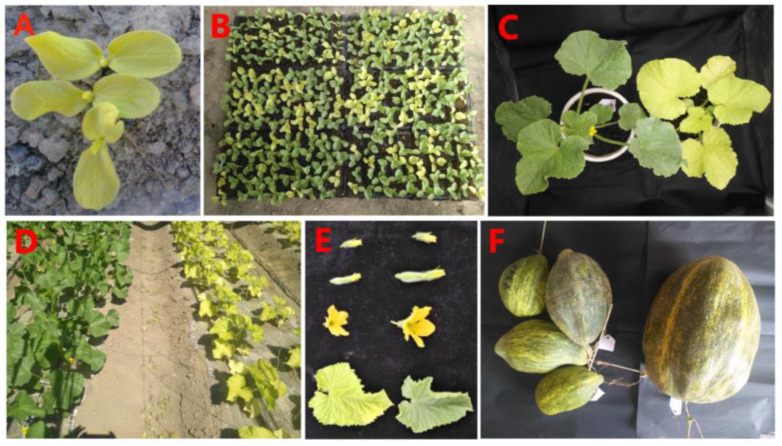
The phenotypic characteristics of yellow-green mutants (MT) and wild-type (WT) plants of Hami melon. (**A**) cotyledon stage of MT; (**B**) F2 progenies; (**C**) seedling stage of MT (right) and WT (left); (**D**) The field performance of MT (right) and WT (left) plants; (**E**) reproductive organs of MT (left) and WT (right); (**F**) Fruits of MT (left) and WT (right) plants.

The MT was obtained from the inbred line ‘Paotaihong’ (a local variety in Xinjiang) (WT) under standard cultivation conditions. The leaves of mutant plants showed slower growth (Supplementary Materials Table S1) and were clearly distinguished by a yellow-green leaf color phenotype throughout the growth period (Figure 1A,C,D). The yellow-green leaf phenotype of MT could be inherited stably after self-crossing, and the leaf color of F1 hybrid (MT×WT) was restored. Compared with WT, the size of flower organ and fruit of MT are smaller (Figure 1E,F). The leaf color of the F2 populations was segregated, with the ratio of 1:3.14 (yellow-green leaves (207 individual plants) to green leaves (649 individual plants)), which accords with the Mendelian law of 1:3 (χ² = 0.305) (Figure 1B). This result indicated that the yellow-green leaf mutant phenotype is a quality trait regulated by a single recessive nuclear gene.

### 2.2. Changes in Chloroplast Ultrastructure and Photosynthetic Pigments

**Figure 2:**
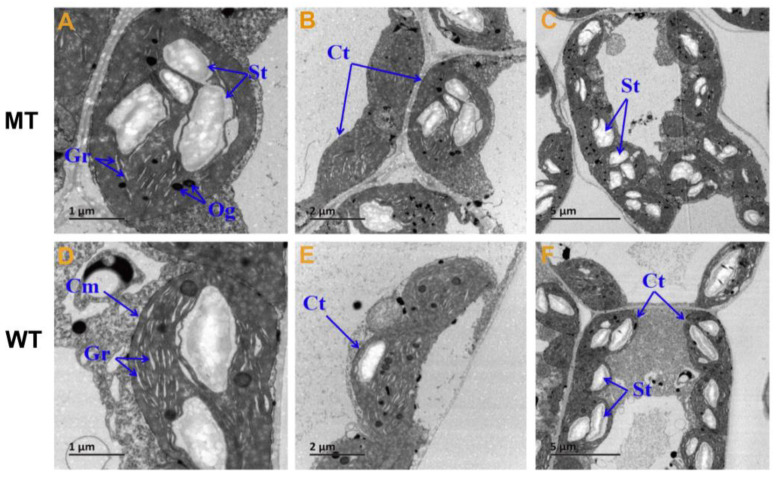
The ultrastructure of chloroplast in MT and WT. (**A**,**D**) Bar = 1 um; (**B**,**E**) Bar = 2 um; (**C**,**F**) Bar = 5 um; Ct, chloroplast; St, starch granules; Gr, grana; Cm, chloroplast outer membrane; Og, osmiophilic globule; MT: the chlorophyll deficiency mutant with yellow-green leaf color; WT: wild type with the normal green leaf color.

**Figure 3:**
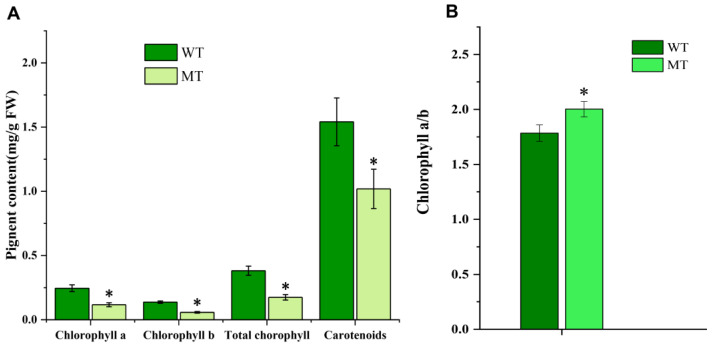
Comparison of photosynthetic pigments in leaves of mutant (MT) and wild-type plants (WT). (**A**) Contents of photosynthetic pigments in leaves of MT and WT; (**B**) the ratio of chlorophyll a to chlorophyll b in MT and WT. * indicates significant differences (*p* < 0.05).

The ultrastructure of chloroplasts and contents of photosynthetic pigments are shown in Figure 2 and Figure 3. The lamella were significantly reduced in number, and were loosely arranged in MT compared with WT (Figure 2), indicating defective structural development of chloroplasts in MT. Chlorophyll a, chlorophyll b, total chlorophyll, and carotenoid contents in MT leaves were all significantly reduced compared with those in WT, whereas the chlorophyll a/b ratio in MT leaves was significantly higher, which indicated that the yellow-green leaf of MT is related to the decreased chlorophyll content, especially chlorophyll b (Figure 3).

### 2.3. The Production and Accumulation of ROS

Defects of chloroplast development are often accompanied by excessive ROS accumulation. Table 1 shows that the contents of MDA and H_2_O_2_ and the generation rate of O_2_^−^ were significantly higher (15.8%, 31.7%, and 387.2%, respectively) in MT than WT. The activity of enzymes (APX, POD, SOD, and CAT) in antioxidant defense system were also significantly increased (60.9%, 16.1%, 7.8% and 7.2%, respectively) in MT. In addition, the content of reduced glutathione (GSH) was increased by 21.8%; however, the oxidized glutathione (GSSG) declined by 44.6% in MT compared with WT. These results indicated that excessive ROS are generated and accumulated in mutants, which induces the oxidative stress response.

### 2.4. Transcriptome Analysis

To further investigate the molecular mechanisms affected by the mutation, we performed transcriptome sequencing on a total of six samples of MT and WT seedlings on the Illumina platform to screen for differentially expressed genes (DEGs). On average, 44,368,761 clean reads were obtained per sample, with an average Q30 of 92.38%. The average percentage of reads that mapped to unique positions on the reference genome was 94.84% (Supplementary Table S2).

#### 2.4.1. Validation of DEGs Transcript Levels

qRT-PCR was used to validate the RNA-seq results. Eighteen DEGs with important functions were subjected to qRT-PCR expression analysis (Supplementary Table S3). Sixteen of these DEGs are involved in carbon metabolism (PCK, PPDK, FBP, TIM, SDHB, ICDH), glycolysis/gluconeogenesis (PCK, PPDK, FBP, TIM, HK1), carbon fixation in photosynthetic organisms (PEP4, FBP, TIM, MDH1), starch and sucrose metabolism (SUS5, TPP, BAM3, UGP1, PWD), and glutathione metabolism (GSHPx, ODC1). The two other genes were CYP98A2 and CoAOMT. The expression of these genes was highly consistent with the RNA-seq data (R^2^ = 0.9211) (Supplementary Figure S1), which indicated the reliability of RNA-seq results.

#### 2.4.2. Gene Ontology (GO) and KEGG Pathways Enrichment Analyses

**Figure 4:**
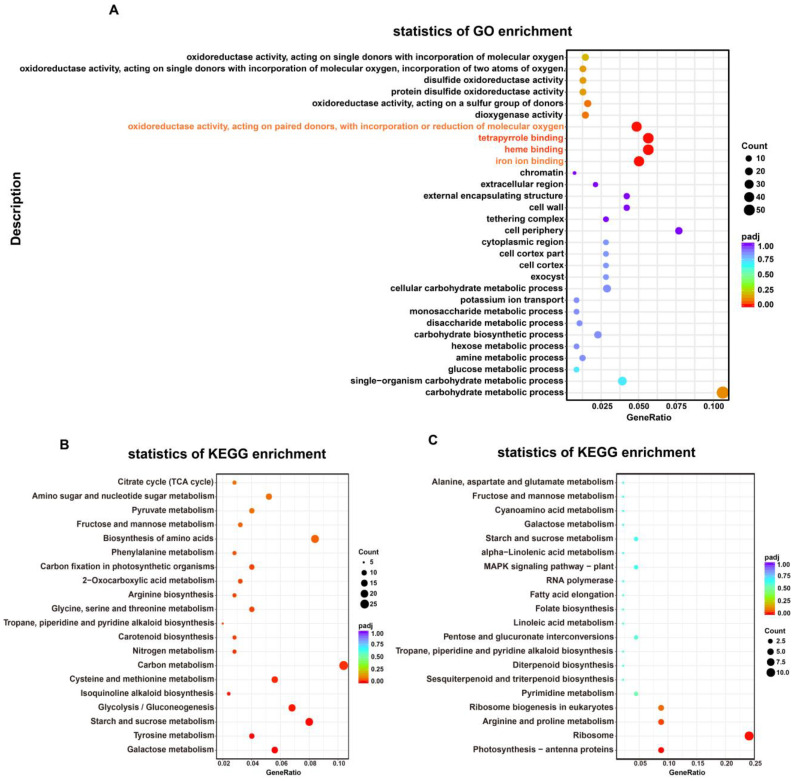
Functional annotation and classification of DEGs identified in MT vs. WT transcriptome, as determined using the GO and KEGG databases. (**A**) GO enrichment analysis; (**B**) KEGG enrichment analysis of up-regulated DEGs; (**C**) KEGG enrichment analysis of down-regulated DEGs. GeneRatio, the ratio between DEGs annotated to a particular KEGG pathway/GO term and all DEGs annotated to KEGG pathway/GO category; padj, *p*-values corrected for multiple hypothesis testing, a lower padj indicates more significant enrichment of the DEGs; count, the number of DEGs annotated to KEGG pathway/GO term. All the analyses used WT as a control.

Using |log2FC| ≥ 1 as a threshold for differential gene expression and a *p*-value < 0.05 as the minimal statistical significance, a total of 1370 DEGs were identified in MT vs. WT leaves; of these, 1140 DEGs were up-regulated and 230 DEGs were down-regulated (Supplementary Figure S2). To further analyze the biological functions of DEGs, we performed GO and KEGG analysis on these DEGs. The gene list and gene number of each term were calculated by using the differential genes annotated by GO term, and the *p*-value was calculated by the hypergeometric distribution method (*p*-value < 0.05 was the standard for significant enrichment) to find the GO term with significant enrichment of differential genes compared with the whole genome background. Thus, the main biological functions performed by the differential genes were determined. The results showed that a total of 1285 DEGs, of which 1057 were up-regulated and 228 were down-regulated, were in different GO categories, namely biological processes (BP) (494), cellular components (CC) (144), and molecular functions (MF) (647). Carbohydrate metabolic processes and single-organism carbohydrate metabolic processes were the most highly enriched GO terms in the BP category. GO MF analysis showed that many DEGs were enriched in oxidoreductase activity terms. In addition, the largest number of DEGs were annotated to heme binding (34), tetrapyrrole binding (34), iron ion binding (30), and oxidoreductase activity (29) (Figure 4A), and most of these were significantly up-regulated in MT compared with WT (Supplementary Figure S3). These results indicated that the redox balance has been significantly affected in MT.

In total, 298 of 1370 DEGs were annotated by the KEGG database. Among these, up-regulated DEGs were significantly enriched in carbon metabolism, and starch and sucrose metabolism. Galactose metabolism, tyrosine metabolism, starch and sucrose metabolism, and glycolysis/gluconeogenesis were the most abundantly enriched terms in the KEGG pathway (Figure 4B). Down-regulated DEGs were significantly enriched (*p* < 0.05) in photosynthesis-antenna proteins, ribosome, and arginine and proline metabolism pathways (Figure 4C).

#### 2.4.3. Functional Prediction of Differentially Expressed Transcription Factors

**Figure 5:**
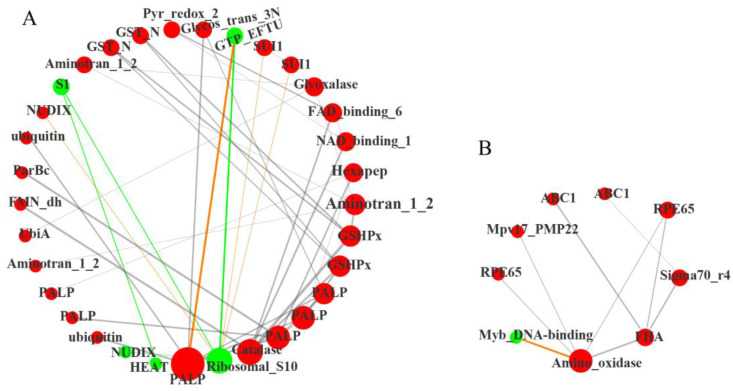
Co-expression network reveals the regulatory of differentially expressed transcription factors (TFs) in MT vs. WT. (**A**) The interaction network of three hub genes (encoding PALP, Catalase and Ribosomal_S10); (**B**) The interaction network of a central gene (encoding Amino_oxidase). The red balls represent the up-regulated expression of TFs in MT, and the green balls represent down-regulated. Orange lines represent negative regulation relationship, gray and green lines represent positive regulation relationship. The size of the node represents the core position of the transcription factor. The width of the connecting line represents the action strength of the corresponding interaction relationship. All the analyses used WT as a control.

Transcription factors (TFs) play a crucial role in plant growth and development. In the current analysis, 86 differentially expressed TFs were identified (Supplementary Table S4). We uploaded these TFs into the STRING database (https://cn.string-db.org/ (accessed on 19 June 2022)) to screen the PPI action pairs containing the differential genes of cucumis melo with Score > 0.95, then obtained the correlation between all the target genes (*ppi.network.txt, Supplementary Table S5) for visual diagram by Cytoscape. The color and size of nodes in the network diagram were adjusted based on the attribute file (Supplementary Table S5). The visualized network diagram showed that the network connected 42 nodes with 104 edges (Figure 5). Figure 5A shows three hub genes, of these, genes encoding PALP (cysteine/tryptophan synthase β-chain, *LOC_103494920*), and Catalase (*LOC_103493812*) were significantly up-regulated, while the gene encoding Ribosomal_S10 (40S ribosomal protein S20-2, *LOC_103504663*) was significantly down-regulated. Ubiquitin (Polyubiquitin protein-like, *LOC_103485541*; Polyubiquitin protein, *LOC_103494931*), Glycos_trans_3N (anthranilate phosphoribosyl transferase, *LOC_103483625*), and GTP_EFTU (EF1 elongation factor 1-alpha, *LOC_103496960*) may directly interact with PALP. Proteins potentially interact with Ribosomal_S10 were NUDIX18 (nudix hydrolase 18, *LOC_103504536*), S1 (RRP5, rRNA biogenesis protein, *LOC_103486130*), GTP_EFTU (*LOC_103496960*), and SUI1 (protein translation initiation factors, *LOC_103490142*, *LOC_103501585*). ParBc (sulfiredoxin, *LOC_103498252*), FMN dh (glycolate oxidase, *LOC_103499183*), FAD_binding_6 (nitrite reductase, *LOC_103487560*), NAD_binding_1 (nitrate reductase, *LOC_103499266*), and GSHPx (glutathione peroxidase, *LOC_103493737*) were related to catalase (CAT) activity; genes encoding these proteins were all significantly up-regulated. These results indicated that the ROS scavenging system regulated by CAT was obviously activated in MT. In addition, the synthesis of proteins in the ribosome may have been severely affected in MT, which may be due to the excess accumulation of ROS.

Figure 5B showed the other central gene encoding Amino_oxidase (lycopene isomerase, *LOC_103486713*) was significantly up-regulated. Except for MYB_DNA binding gene (MYB34-like, *LOC_103484167*), genes encoding proteins directly related to Amino_oxidase were all significantly up-regulated, including the Amino_oxidase (lycopene isomerase, *LOC_103486713*), RPE65 (carotenoid 9,10 (9’, 10’)-cleavage dioxygenase 1 (CCD1), *LOC_103499930*; carotenoid cleavage dioxygenase 4 (CCD4), *LOC_103493037*), and FHA (zeaxanthin epoxidase (ZEP), *LOC_103497391*), which were closely related to carotenoid metabolism. These indicated that the degradation of carotenoids might be mobilized to protect photosynthetic organs from excessive ROS in MT.

### 2.5. Metabolomic Analysis

**Figure 6:**
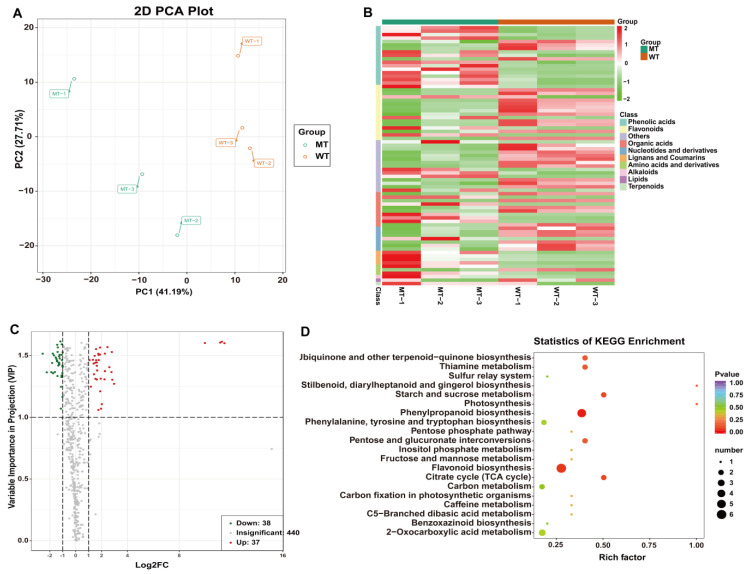
KEGG classification statistics and enrichment analysis of DAMs in MT vs. WT. (**A**) MT VS WT opls score: in the x-coordinate, P1 represents the predicted principal component and O1 represents the orthogonal principal component; (**B**) KEGG classification statistics of DAMs in MT vs. WT; (**C**) Differential metabolite volcano map; (**D**) Scatter plot of DAMs enriched KEGG pathways. Rich factor is the ratio of the DAM number to the background number in a certain pathway. The size of the dots represents the number of metabolites, and the color of the dots represents the range of the *p*-value.

To clarify changes in the metabolic levels of MT and WT, we used a total of six biological replicates of MT and WT seedling leaves for qualitative and quantitative analyses of metabolites by using ultra-performance liquid chromatography-tandem mass spectrometry (UPLC-MS/MS). A total of 515 metabolites were detected, 255 of which were mapped onto the known metabolic pathways. Principal component analysis (PCA) revealed significant differences in metabolites between MT and WT samples. Partial least squares discriminant analysis (PLS-DA) and orthogonal partial least squares discriminant analysis (OPLS-DA) were performed on the metabolic profiles (Figure 6A). Screening for significantly different metabolites was performed with projection variable importance (VIP) > 1 and fold change ≥ 2 or fold change ≤ 0.5. A total of 75 differential accumulation metabolites (DAMs) were detected; 38 were elevated and 37 of them were reduced in MT compared with WT (Figure 6C). KEGG Classification analysis showed that 38 out of 75 DAMs were enriched in 37 metabolic pathways, with a large number of DAMs enriched in phenylpropanoid (4) and flavonoid biosynthesis (5) pathways (Supplementary Figure S4). Photosystem, stilbenoid, diarylheptanoid and gingerol biosynthesis, starch and sucrose metabolism, citrate cycle (TCA), ubiquinone terpenoid-quinone biosynthesis, thiamine metabolism, and pentose-glucuronide conversion pathway were the most highly enriched terms in the KEGG pathway, according to their enrichment factor (Figure 6D).

### 2.6. Key Gene Scramble between Metabolism and Transcriptional Levels

**Figure 7:**
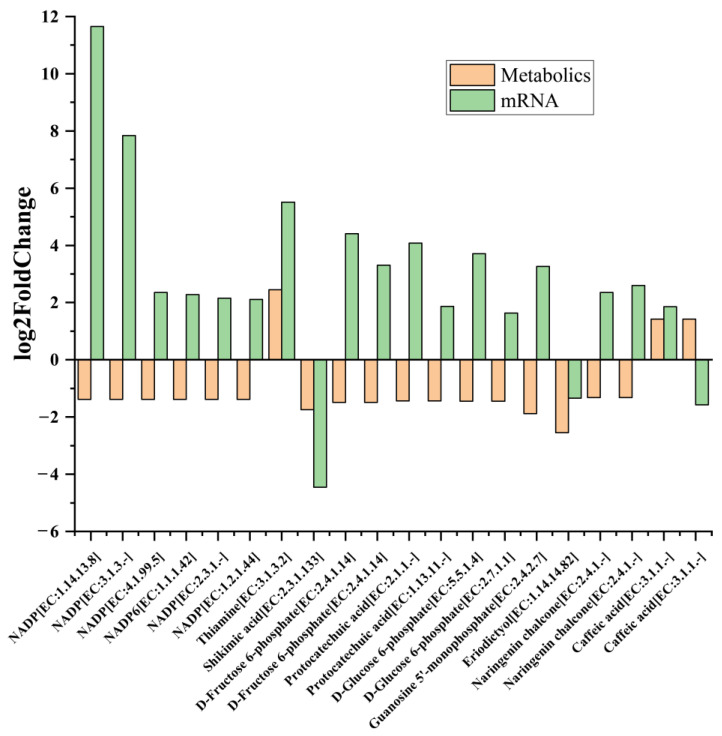
The log2FoldChange of top 20 pairs DAMs and DEGs in co-enriched metabolic pathways (*p* < 0.05) with the most obvious difference in MT vs. WT. The analyses used WT as a control.

The DAMs and DEGs (*p* < 0.05) were simultaneously mapped to the KEGG pathway database to obtain common pathway information, and these results were visualized using the R path View package. Figure 7 demonstrates the top 20 in log2FC of DAMs and DEGs in co-enriched metabolic pathways (*p* < 0.05) with the most obvious difference in MT vs. WT. The top DAMs obtained from this analysis were nicotinamide adenine dinucleotide phosphate (NADP^+^), D-fructose-6-phosphate, protocatechuic acid, D-Glucose-6-phosphate, thiamine, shikimic acid, guanosine 5′-monophosphate, caffeic acid, naringenin chalcone, and eriodictyol. All of these were down-regulated, except for thiamine and caffeic acid. These metabolites and enzyme genes are mainly involved in photosynthesis, central carbon metabolism and energy metabolism, glutathione metabolism, phenylpropanoid biosynthesis, and flavonoid metabolism. Therefore, further analysis of the transcriptome and metabolomics data was conducted around these pathways.

**Figure 8:**
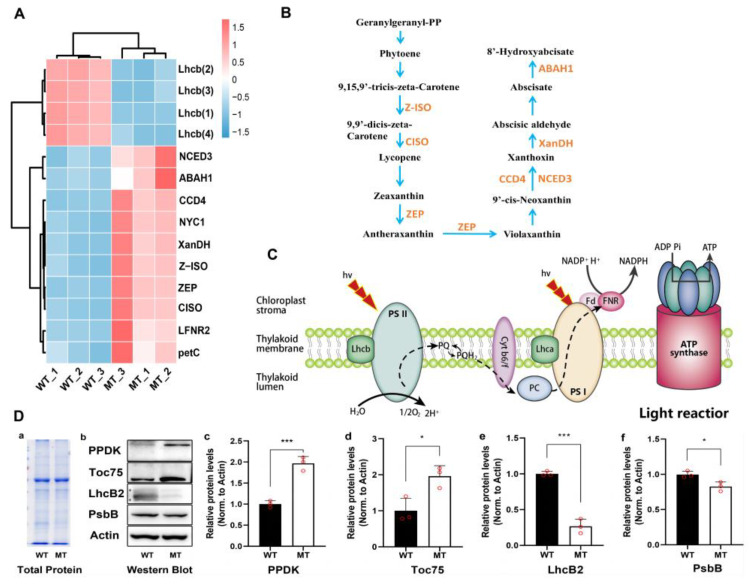
Analysis of DAMs, DEGs and DAPs in the metabolic pathways related to photosynthesis. (**A**) Relative expression level of DEGs in light reactions and carotenoid biosynthesis. Lhcb, light-harvesting complex of PSII; LFNR2, ferredoxin--NADP reductase leaf isozyme; petC, cytochrome b6-f complex iron-sulfur subunit. NYC1, chlorophyll(ide) b reductase; CCD4, carotenoid cleavage dioxygenase 4; ZEP, zeaxanthin epoxidase; XanDH, xanthoxin dehydrogenase; Z-ISO, 15-cis-zeta-carotene isomerase; NCED3, 9-cis-epoxycarotenoid dioxygenase; ABAH1, abscisic acid 8′-hydroxylase 1-like; CISO, prolycopene isomerase. (**B**) Carotenoid metabolism pathway. (**C**) Light reaction stage. Lhcb, light harvesting complex of PSII; PQ, plastoquinone; Cyt b6/f, cytochrome b6/f; PC, plastocyanin; Lhca, light harvesting complex of PSⅠ; Fd, ferredoxin; FNR, ferredoxin-NADP+ reductase. (**D**) Relative quantitative analysis of proteins. a, total protein gel staining map; b, western Blot diagram; c, quantitative results of PPDK (pyruvate phosphate dikinase); d, quantitative results of Toc75 (chloroplast outer envelope mebrane translocon complex OEP75 protein); e, quantitative results of of LhcB2 (LHCII type chlorophyll a/b-binding protein); f, quantitative results of PsbB (PSII CP47 reaction center protein). All the analyses used WT as a control, and relative quantitative analysis of proteins using Actin protein as internal reference. * and *** indicate significant differences (0.01 < *p* <  0.05, *p* < 0.001).

DEGs and DAMs involved in photosynthesis-antenna proteins, porphyrin and chlorophyll metabolism, and carotenoids metabolism were analyzed (Figure 8). The four LhcB-like1 genes encoding the peripheral light-harvesting antenna protein component (LhcB) in PSII were significantly down-regulated in MT. The genes encoding ferredoxin-NADP reductase leaf isozyme (LFNR2) and cytochrome b6-f complex iron-sulfur subunit PetC) were significantly up-regulated. Additionally, the chlorophyll (ide) b reductase NYC1 gene (*LOC_103501561*) involved in porphyrin and chlorophyll metabolism, and seven genes encoding key enzymes of the carotenoids biosynthesis pathway, such as CCD4, ZEP, XanDH, Z-ISO, NCED3, ABAH1, and CISO were all significantly up-regulated in MT (Figure 8A,B).

Compound NADP+, the final electron receptor in photosynthetic electron transport chain (ETC), was significantly reduced in MT (Figure 7 and Figure 8C), which may result in the obstruction of electron transfer or electronic leakage. To investigate the consequence of reduced expression of photosynthetic genes, Western blot analysis was employed (Figure 8D). Whereas the overall proteome patterns were not significantly different in MT vs. WT, proteins levels of LhcB2 and PsbB (chlorophyll a binding protein of PSII) were significantly reduced in MT. By contrast, the levels of Toc75 (chloroplast protein transport channel protein) and PPDK (pyruvate phosphate dikinase) were significantly increased in MT compared with WT. These above changes might affect the photosystem activity and ETC electron transfer efficiency, and the yellow-green leaf phenotype of MT.

### 2.7. Carbohydrate Metabolism and Energy Metabolism

**Figure 9:**
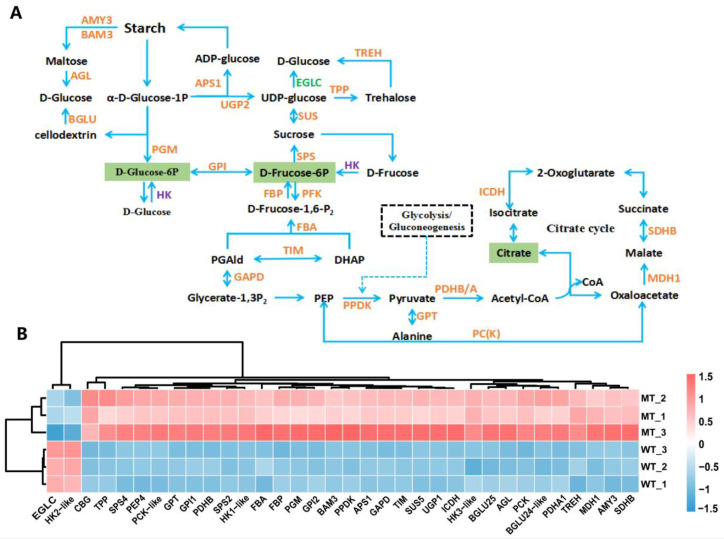
Central carbon metabolism pathway (**A**) and relative expression level of DEGs (**B**). CBG, cyanogenic beta-glucosidase-like(Glyco_hydro_1); SPS4, sucrose-phosphate synthase; BGLU, beta-glucosidase; TPP, trehalose-phosphate phosphatase; SUS, sucrose synthase; TREH, trehalase; HK, hexokinase; AMY, alpha-amylase; GPI, glucose-6-phosphate isomerase; UGP, UTP-glucose-1-phosphate uridylyltransferase; BAM, beta-amylase; PGM, phosphoglucomutase; APS1, glucose-1-phosphate adenylyltransferase small subunit; AGL, 4-alpha-glucanotransferase DPE2; PCK, phosphoenolpyruvate carboxykinase [ATP]; GPT/ALT, alanine aminotransferase; PEP4, phosphoenolpyruvate carboxylase 4; GAPD, glyceraldehyde-3-phosphate dehydrogenase; TIM, triosephosphate isomerase; FBP, fructose-1,6-bisphosphatase; FBA, fructose-bisphosphate aldolase cytoplasmic isozyme; PPDK, pyruvate phosphate dikinase; PDHB/A, pyruvate dehydrogenase E1 component subunit beta/alpha; ICDH, cytosolic isocitrate dehydrogenase [NADP]; MDH1, malate dehydrogenase 1; SDHB, succinate dehydrogenase [ubiquinone] iron-sulfur subunit; EGLC, glucan 1,3-beta-glucosidase. The analyses used WT as a control.

As shown in Figure 9, nearly all of the DEGs involved in carbohydrate metabolism and energy metabolism were significantly up-regulated in MT, such as fructokinase 6-phosphate kinase 6 (PFK6) gene, fructose-1,6-bisphosphatase (FBP) gene, triose phosphate isomerase (TIM) gene, raw cyanate β-glucosidase (CBG) gene, glucose-6-phosphate isomerase (GPI) gene, galactitol-sucrose galactosyltransferase 5 (RFS5) gene, galactitol synthase 2 (GOLS2) gene, hexokinase 1/3 (HK1/3) genes and so on, although the hexokinase-like2 (HK-like2) and glucan endo-1,3beta-glucosidase (EGLC) genes were down-regulated (Figure 9B). These genes encode key enzymes in various pathways such as galactose metabolism, glycolysis/gluconeogenesis, starch and sucrose metabolism, citrate cycle (TCA cycle), and carbon fixation in photosynthetic organisms. Compounds such as D-fructose 6-phosphate, D-glucose 6-phosphate, and citric acid, which are key intermediate metabolites of the aforementioned pathways, showed reduced accumulation in MT (Figure 9A). These results suggested that the carbon and energy metabolism was strongly affected in MT.

### 2.8. Metabolic Pathways Related to Redox Balance

**Figure 10:**
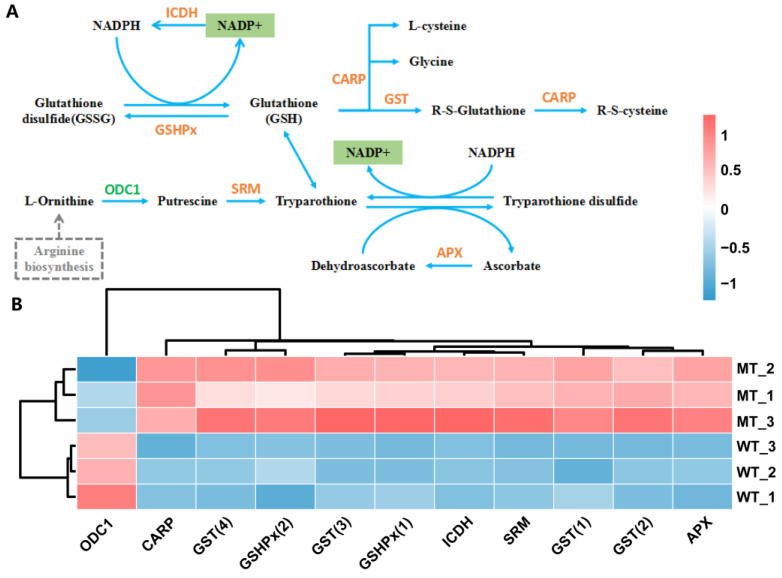
Glutathione metabolism pathway (**A**) and relative expression level of DEGs (**B**). GST, glutathione S-transferase; CARP, leucine aminopeptidase; ICDH, cytosolic isocitrate dehydrogenase [NADP]; APX, L-ascorbate peroxidase; GSHPx, phospholipid hydroperoxide glutathione peroxidase; SRM/SPE, spermidine synthase; ODC1, ornithine decarboxylase. The analyses used WT as a control.

NADP+, which is an integral part of the redox mechanisms such as the glutathione metabolism pathway (ko00480), was significantly reduced in MT (Figure 10A). As shown in Figure 10B, except for the ornithine decarboxylase (ODC1) gene, all of the DEGs related to the glutathione metabolic pathway were significantly up-regulated in MT as compared with those in WT, such as L-ascorbate peroxidase (APX) gene, leucyl aminopeptidase (CARP) gene, spermidine synthase (SRM) gene, glutathione peroxidase (GSHPx) gene, cytosolic isocitrate dehydrogenase (ICDH) gene, and glutathione S-transferase (GST) gene.

**Figure 11:**
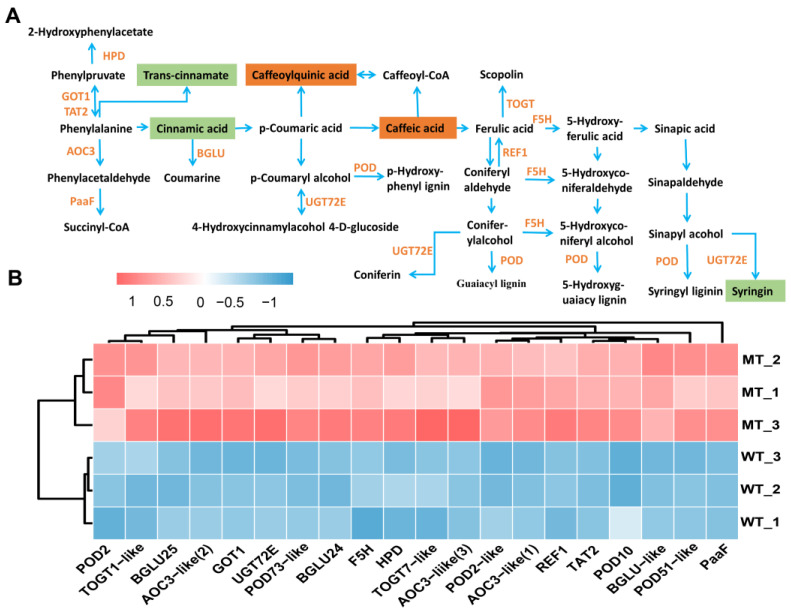
Phenylalanine metabolism and phenylpropanoid biosynthesis pathway (**A**) and relative expression level of DEGs (**B**). POD, peroxidase; F5H, cytochrome P450 84A1; BGLU, beta-glucosidase; REF1, aldehyde dehydrogenase; UGT72E, anthocyanidin 3-O-glucosyltransferase; TOGT1-like, scopoletin glucosyltransferase1-like; TOGT7-like, UDP-glucose flavonoid 3-O-glucosyltransferase 7-like; TAT2, probable aminotransferase; AOC3-like, primary amine oxidase-like; GOT1, aspartate aminotransferase; PaaF, probable enoyl-CoA hydratase; HPD, 4-hydroxyphenylpyruvate dioxygenase. The analyses used WT as a control.

Phenylpropanoid metabolism provides plants with flavonoids that scavenge ROS induced by environmental stresses such as UV-B, high salinity, drought, and extreme temperatures (Nakabayashi and Saito 2015; Sharma et al., 2019). In the present study, metabolomic results showed that 12 out of 75 DAMs were enriched in phenylalanine, tyrosine and tryptophan biosynthesis, phenylpropanoid biosynthesis and flavonoid biosynthesis pathways. Metabolites such as cinnamic acid, syringin, eriodictyol, naringenin chalcone, pinobanksin, and butin showed significantly reduced accumulation, whereas chlorogenic acid, hydroxy-methoxycinnamate, caffeic acid, and hesperetin O-malonylhexoside showed significantly increased accumulation in MT (Figure 11A). DEGs enriched in phenylalanine metabolism and phenylpropanoid biosynthesis, which are secondary metabolic pathways related to plant defense under stress, were all significantly up-regulated (Figure 11B). These results suggested that the glutathione metabolism, phenylpropanoid and flavonoid metabolism were all significantly altered to deal with the ROS-induced oxidative stress in MT.

## 3. Discussion

### 3.1. Changes in Photosystem Cause Leaf Yellowish Phenotype in Mutant

Biosynthesis and breakdown of chlorophyll are tightly regulated throughout the plant development [49,50]. For example, losing green color with decreased chlorophyll amount is a biomarker of leaf senescence [51]. As the major antenna protein complexes that bind chlorophyll a/b in PSII, LHCIIs account for nearly 50% of the pigments in the photosynthetic membranes and nearly 30% of the total protein in the chloroplast membranes [52,53,54,55]. In Arabidopsis, three major isoforms (LhcB1, LhcB2 and LhcB3) of LHCIIs work in a trimeric form. These isoforms present in different molar ratios and each monomer could bind eight chlorophyll a, six chlorophyll b, and four carotenoids. The ratio of LhcB is not constant but varies depending on the light conditions [33,56,57]. In our results, the higher ratio of chlorophyll a to chlorophyll b in mutants is probably due to chlorophyll b degradation (Figure 3B), although either chlorophyll a or chlorophyll b decreased obviously. Accordingly, we also found that both LhcB transcript expression and LhcB2 protein were reduced (Figure 8). These results agreed with the report that LHCII degradation can be induced by chlorophyll b degradation [57]. Beyond that, throughout the pathway of chlorophyll metabolism, chlorophyll b can be catalyzed by NYC1 in the very first step into chlorophyll a and further used in antenna complex formation in PSⅠ and PSII [57,58,59]. In the present study, we found the NYC1 transcript expression in mutant was up-regulated (Figure 8A), which was in accordance with the chlorophyll b degradation. It was proved that natural or dark-induced senescence enhanced NYC1 expression and further promoted chlorophyll b degradation [57]. The results suggest that the decreased chlorophylls, especially chlorophyll b, give the MT leaf a uniformly yellowish color and induce NYC1 up-regulation and LhcB degradation.

In addition, thylakoids in chloroplasts are enriched with constituents of PSI and PSII. Research has shown that PSII and LHCIIs mainly locate at the granal thylakoids, PSI and ATPase locate at the stromal thylakoids, and cytochrome b6/f complex exists in between these two compartments [33,56,60]. Plants are always able to carry out efficient repairment of PSII through modulating thylakoid membrane structure, such as the proportion of granal thylakoids relative to stroma thylakoids [61,62]. Here, we observed the thylakoid grana stacks in mutants were reduced and loosely arranged (Figure 2), which was obviously different from WT. Also, the transcript expression level of two key genes in PSI, LFNR2 and petC, were also significantly up-regulated in mutants in response to the shortage of substrates NADP^+^ from PSII. In maize, the LFNR2 transcript abundance was proposed to be regulated strongly dependent on the photosynthetic state [63,64]. In addition, protein level of Toc75, the channel for importing nucleus-encoded chloroplast proteins across the chloroplast’s outer envelope membrane [65,66], was significantly elevated in MT (Figure 8D). This may be an adaptation regulation in response to the reduced chloroplast protein levels, such as LhcB2 and PsbB, by promoting the import efficiency of such nucleus-encoded chloroplast proteins. These data demonstrate that under the circumstances of PSII deficiency, regulations in the photosystem and/or the following operations enable the mutant to adapt and survive.

### 3.2. NADP^+^ Deficiency Resulted in Excessive Accumulation of ROS in Mutant

ROS play an integral role as plant signaling molecules in the regulation of many biological processes, such as plant growth and development, and response to biotic or abiotic stresses [67]. Under normal physiological conditions, cellular ROS levels are stable as a result of dynamic equilibrium of ROS production and elimination [68]. However, when plants are subjected to stress or abnormal oxidation, substantial accumulation of ROS occurs [69], which in turn impedes protein synthesis by altering basic cellular properties such as membrane fluidity and ion transport. It also causes enzyme inactivation, protein cross-linking, DNA damage, pigment decolorization, protein degradation, and lipid peroxidation [70]. POD, SOD, CAT, APX and the AsA-GSH cycle are the antioxidant systems that have evolved for efficient ROS scavenging in plants. In our previous research, although the activity of SOD, APX, POD, and CAT, and the higher ratio of GSH/GSSH significantly increased in MT compared with WT, the contents of malondialdehyde (MDA), superoxide anion radicals (O_2_^−^), and hydrogen peroxide (H_2_O_2_) were significantly increased in MT (Table 1). In addition, the expression of catalase gene and its interacting TFs were all significantly up-regulated in MT (Figure 5). These results indicate that the mutation has resulted in excessive generation and accumulation of ROS in MT, which disrupted the redox balance and induced a series of oxidative stress responses.

Chloroplast is one of the primary sources of ROS in plant cells, along with the mitochondria and peroxisomes. The rapid energy transfer during photon capturing, electron transfer, and oxidation-reduction potential in photosynthesis, leads to the release of ROS, including singlet oxygen (^1^O_2_), O_2_^−^, and H_2_O_2_ [71]. NADP^+^ is a key electron carrier for redox reactions including photosynthesis, mediating the conversion of photosynthetic energy as the final electron acceptor in photosynthesis, and promoting anabolism to support plant growth [72,73]. Normally, electrons flow from the active PS centre to NADP^+^, reducing it into NADPH, which then enters the Calvin cycle and assimilates CO_2_ to synthesis organic compounds. When the photosynthetic fixation of CO_2_ was limited, the utilization of NADPH was decreased, with a resultant decline in the level of NADP^+^ [74]. Given that NADP^+^ is a major acceptor of electrons in PSI, depletion of NADP^+^ triggers the transport of electrons from PSI to molecular oxygen, which generates ROS [75]. Our previous study on the induced kinetics of chlorophyll fluorescence showed that less energy absorbed by photosynthetic reaction center is used for electron transfer and more is dissipated through photorespiration in MT compared with WT [76]. This indicates that the fixation of CO_2_ in the Calvin cycle is limited, which results in significantly declined level of NADP^+^ in MT (Figure 6). Elevated expression of genes encoding ferredoxin-NADP reductase leaf isozyme (LFNR2) and cytochrome b6-f complex iron-sulfur subunit (petC) in the electron transport chain can be interpreted as a feedback adjustment to cope with insufficient supply of NADP^+^ in MT (Figure 8A). In short, the generation and accumulation of excess ROS might be mainly caused by insufficient NADP^+^, which may be related to the limitation of the carbon fixation in the Calvin cycle in MT.

### 3.3. ROS Acts as a Signal Molecule for Feedback Regulation of Chloroplast Development

The available information suggests that H_2_O_2_ produced in the chloroplasts under high light conditions can diffuse from the chloroplast to the cytoplasm and interact with the abscisic acid (ABA) signaling network to down-regulate the expression of LhcB genes [77,78]. Carotenoids are essential components of the plant light absorption complex and play an important role not only in protecting photosynthetic organs from photo-oxidative damage but also as the precursors for the synthesis of ABA. In this study, although the carotenoid content declined significantly in MT, seven DEGs enriched in the carotenoids metabolic pathway were significantly up-regulated in MT (Figure 8A,B), including 9-cis-epoxycarotenoid dioxygenase gene (NCED3), which catalyzes cleaving 9-cis xanthophylls to xanthoxin (a precursor of ABA), the key regulated step in ABA biosynthesis in plants [79,80]. These results indicate that the synthesis of ABA may be promoted in MT.

In short, the excessive accumulation of ROS might interact with ABA signaling network to down-regulate LhcB to eliminate chlorophyll b and LHCII proteins in MT. These may result in the inhibition of chloroplast protein synthesis and assembly, and the decline of chlorophyll content. These could also be regarded as an adaptive measure to overcome the disrupted photosynthetic electron transport in MT, that is, reducing photosynthetic electron input by reducing light energy capture to alleviate photo-oxidative damage due to excess light energy.

### 3.4. The Abnormal Carbon Metabolism Limited Growth and Development in Mutants

Plants, as sequestered organisms, are affected by changing environments and frequent biotic and abiotic stresses, which induce reallocation of energy and carbon fluxes to adapt to the changing environment and initiate specific signaling pathways to cope with the ensuing damage [81]. Sugar is the main carbon and energy source in cells, and effects on plant growth and development have often been attributed to sugar metabolism [82]. However, recent evidence has shown that sugars can also act as signaling molecules that affect the whole plant life cycle [83]. In plants, the regulatory effects of glucose have been the most studied to date. Hexokinase (HK), the first Glc sensor identified in plants, is not only involved in sugar metabolism relying on hexose phosphorylation function but also acts as a hexose sensor and signal to sense external nutrients, light and hormones, and regulate plant growth in signaling networks [83,84,85,86].

In this study, a total of 34 DEGs involved in starch and sucrose metabolism, carbon fixation in photosynthetic organisms, and citrate cycle were significantly up-regulated in MT, including HK1/3. By contrast, D-Glucose 6-phosphate and D-fructose-6-phosphate, the derivate metabolites of Glc, were shown to markedly decline in MT compared with WT (Figure 9). Given that Glc and fructose are the raw material for most of the metabolic pathways and organic matter in plants [87], it can be speculated that significant changes in carbon and energy metabolism occur in MT. The significantly up-regulated expression of HK1/3 in MT compared with MT, might function in the feedback regulation in response to reduced D-Glucose 6-phosphate and D-fructose-6-phosphate. HKs act as Glc sensors that regulate the activity of photosynthesis through the repression of several photosynthetic genes, such as LHCB (formerly known as chlorophyll a/b-binding proteins, CAB) genes [88]. Therefore, the significantly up-regulated expression of HK1/3 may be also responsible for the inhibition of photosynthesis in MT. It is intriguing that the HK2 gene was significantly down-regulated in MT, suggesting that the HKs family of genes may play different roles in the regulation of plant growth and development.

### 3.5. Adaptive Regulation of Photosynthetic Carbon Assimilation Pathway in MT

The C_4_ photosynthetic pathway is an adaptation derived from the more common C_3_ photosynthetic pathway that confers a higher productivity under warm temperature and low atmospheric CO_2_ concentration [89,90,91]. Evolution of the C_4_ pathway has been seen as a consequence of adaptive shift to adverse environmental conditions such as high temperature, intense light, and drought [92,93]. Although the Kranz structure of C_4_ plants is essential for efficient carbon assimilation, it has been shown that C_3_ plants without the typical Kranz structure can also complete the C_4_ photosynthetic carbon assimilation process through the high expression of C_4_ photosynthetic enzymes and their compartmentalized distribution within the chloroplasts [94,95]. This suggested that changes in the C_4_ photosynthetic enzyme activity are evolutionarily critical. This provides a theoretical possibility for improving the photosynthetic efficiency of C_3_ crops by importing C_4_ photosynthetic enzyme genes.

Interestingly, in this study, gene expression and functional analysis showed that the ribulose bisphosphate carboxylase/oxygenase activase gene (RCA), and some of the DEGs involved in the C4-dicarboxylic acid cycle and the CAM cycle, including the phosphoenolpyruvate carboxykinase gene (PCK), phosphoenolpyruvate carboxylase 4 gene (PEP4), PPDK, and the malate dehydrogenase 1 gene (MDH1), were all significantly up-regulated in MT (Figure 9). Western blot also confirmed accumulation of PPDK protein in MT (Figure 8D). These were consistent with our previous research results, namely that the MT has higher activity of key enzymes involved in carbon fixation in photosynthetic pathways, and higher net photosynthetic rate (Pn) at low CO_2_ concentration compared with WT [48]. From these results, we speculated that it may be an adaptive regulation of the reduced photosynthetic carbon assimilation efficiency of the Calvin cycle in MT due to mutation, that is, compensating for the lack of photosynthetic carbon assimilation capacity by improving CO_2_ fixation efficiency through increasing the activity of key enzymes.

The cultivation area of Hami melon is gradually increasing in China, especially by protected cultivation to extend the harvest period. However, insufficient light and CO_2_ in greenhouses has seriously affected the quality of Hami melons. There is no doubt that crops with C_4_ photosynthetic pathway have more advantages in greenhouse cultivation. Although both C_3_ and C_4_ photosynthetic pathways exist simultaneously (Rowan, 2004), the role of the C_4_ pathway has not been fully recognized in C_3_ crops. In Hami melon, as a C_3_ plant, the key enzyme genes involved in C_4_ photosynthetic pathway were significantly up-regulated induced by mutation, in spite of the plants’ weaker growth. Further analyzing this regulation mechanism will be of great significance to breeding with high photosynthetic efficiency.

## 4. Materials and Methods

### 4.1. Plant Materials

A Hami melon (*Cucumis melo* L.) yellow-green leaf spontaneous mutant (MT) was isolated from the wild type ‘paotaihong’ (WT), a local Hami melon variety in Xinjiang, under standard cultivation conditions. Both MT and WT were used for physiological indicators determination, transcriptome sequencing and metabolic spectrum detection. The F_2_ populations, which had 856 individuals, were generated from self-crossing of F1 hybrid (MT×MT) and were used for analysis of genetic characteristics. All materials were cultivated in a solar greenhouse at comprehensive test station of Xinjiang Academy of Agricultural Sciences (87°47′ E, 43°95′ N), Urumqi City, Xinjiang Province, China. The temperature was controlled at 32/20 °C (day/night, 12 h/12 h), the humidity was approximately 50% during the day and 80% at night, and the light intensity was approximately 600 µmol·photons·m^−2^·s^−1^ at noon.

### 4.2. Photosynthetic Pigments Determination

For pigments extraction, the second leaves (from the top) of MT and WT plants were collected comprising about 30 mg of fresh weight at the five-leaf stage. Chlorophyll (Chl) was extracted with 80% (*v*/*v*) acetone and ethanol mixture solution at 25 °C in dark for 48 h until leaves turned white. Chlorophyll a (Chl a), chlorophyll b (Chl b) and carotenoid (Car) were measured with a UV-spectrophotometer (AQ8100, Thermo Orion), according to the method outlined by Arnon (1949) [96].

### 4.3. Transmission Electron Microscopy Analysis

The collected fresh leaves were sliced into 1 mm^2^ sections, then fixed with 2.5% (*w*/*v*) glutaraldehyde for 12 h at 4 °C and washed with 0.1 M sodium phosphate buffer solution (PBS) pH 7.0 three times, 15 min each. Samples were post-fixed with 1% osmium tetroxide for 2 h, and then dehydrated in a graded ethanol series (30, 50, 70, 80, 90, 95, and 100% for 1 h each), and embedded in epon resin. Thin sections stained with toluidine blue were examined by light microscopy, and ultrathin sections obtained with a diamond knife were stained with uranyl acetate and lead citrate [97]. These were observed and photographed using a Jeol 100CX transmission electron microscope (JEOL Ltd., Tokyo, Japan).

### 4.4. Enzyme Activity of Antioxidant Defense System and ROS Contents Determination

To evaluate the redox status of mutants, analysis and quantification of ROS (H_2_O_2_ and O_2_^−^) production in leaves of MT and WT plants were carried out as described previously [98,99]. The contents of malondialdehyde (MDA), glutathione (GSH/GSSG), and the enzyme activity of the antioxidant defense system, including peroxidase (POD), superoxide dismutase (SOD), catalase (CAT), and ascorbate peroxidase (APX), were measured with a UV-spectrophotometer (AQ8100, Thermo Scientific Orion, Waltham, MA, USA), following the instructions of the corresponding kit (Suzhou Keming Biotechnology Co., Ltd., Suzhou, Jiangsu Province, China).

### 4.5. Transcriptome Analysis

At seedling stage, the young leaves (the 2nd spreading leaf from growing point) were collected and immediately stored in liquid nitrogen. Three biological replicates, a total of six samples were used for RNA sequencing. Total RNA was extracted with TRIzol reagent (Thermo Fisher Scientific, Waltham, MA, USA). RNA integrity was assessed using the RNA Nano 6000 Assay Kit of the Bioanalyzer 2100 system (Agilent Technologies, Palo Alto, CA, USA) and RNA purity was checked using the NanoPhotometer^®^ spectrophotometer (IMPLEN, Los Angeles, CA, USA). Six mRNA libraries were generated using NEBNext^®^ UltraTM RNA Library Prep Kit for Illumina^®^ (NEB, Ipswich, MA, USA) following manufacturer’s recommendations and sequenced on an Illumina Novaseq 6000 System (San Diego, CA, USA);150 bp paired-end reads were generated. 

Clean data (clean reads) were obtained by removing reads containing adapter, reads containing ploy-N and low quality reads from raw data. At the same time, the Q20, Q30 and GC contents of the clean data were calculated. The clean reads were aligned to the reference genome (melon (DHL92), ASM31304v1) using HISAT2 (v2.1.0) and transcripts were assembled using assembled by StringTie (v1.3.3b) in a reference-based approach. Then, differential expression analysis across samples was conducted in the edgeR package (http://www.r-project.org/ (accessed on 19 June 2022)) to obtain DEGs (corrected *p* < 0.05, |log2FC| > 1). Gene Ontology (GO) enrichment analysis and KEGG enrichment analysis of differentially expressed genes were implemented by the clusterProfiler R package; we used corrected *p* < 0.05 as a threshold to identify the significant functional categories and metabolic pathways.

### 4.6. RNA Isolation and Quantitative Real-Time PCR

The expression patterns of 18 DEGs with important functions were validated using qRT-PCR (Supplementary Table S3). Every group included three biological replicates. PrimeScript TM 1st stand cDNA Synthesis Kit (TaKaRa Code: D350A) was used to synthesize the cDNAs. The real-time quantification was performed using AceQ^®^ qPCR SYBR^®^ Green Master (Vazyme, Nanjing, Jiangsu Province, China). PCR cycling was performed using a program of 95 °C for 10 min, and 40 cycles of 95 °C for 15 s and 60 °C for 30 s. Actin gene (forward: GGCAGTGGTGGTGAACATG and reverse: TTCTGGTGATGGTGTGAGTC) was used as an internal control gene.

### 4.7. Metabolite Profiling

Metabolite profiling was carried out using a widely targeted metabolome method at Wuhan Metware Biotechnology Co., Ltd. (Wuhan, China) (http://www.metware.cn/, accessed on 18 June 2022). The freeze-dried leaf was crushed using a mixer mill (MM 400, Retsch) with a zirconia bead for 1.5 min at 30 Hz. 100 mg sample powder was weighted and extracted overnight at 4 °C with 0.6 mL 70% aqueous methanol, then adsorbed and filtrated before analysis using a LC-electrospray ionization (ESI)-MS/MS system. We combined the OPLS-DA of the VIP values and the univariate statistical analysis of the fold change to screen for significant differential metabolites between the different comparison groups. Significantly regulated metabolites between groups were determined by VIP ≥ 1 and absolute Log2FC ≥ 1. Identified metabolites were annotated using KEGG Compound database (http://www.kegg.jp/kegg/compound/, accessed on 19 June 2022), annotated metabolites were then mapped to KEGG Pathway database (http://www.kegg.jp/kegg/pathway.html, accessed on 19 June 2022). Pathways with significantly regulated metabolites mapped to were then fed into MSEA (metabolite sets enrichment analysis), their significance was determined by hypergeometric test’s *p* values.

### 4.8. Quantitative Detection of Key Proteins by Western Blot

MT and WT leaf protein extraction was conducted following a procedure similar to that described previously for tomato [66]. Approximately 20 mg leaf tissue was used for each sample, and only leaf lamina tissue was collected to avoid the thick midvein.

For SDS-PAGE, immunoblotting and quantification thereof, procedures were as previously described [66]. Total protein samples of 10 to 20 μg, prepared from melon leaf, were typically analyzed. Primary antibodies anti-Toc75-III (TOC, 75 kD, self-provided), anti-PPDK (PAB07103, Orizymes), anti-PsbB (PAB02004, Orizymes), anti-LhcB2 (PAB06002, Orizymes) and anti-Actin (matched group, LF208, Epizyme), were obtained from Orizymes. The secondary antibody was anti-rabbit immunoglobulin G conjugated with horseradish peroxidase (Santa Cruz Biotechnology (Shanghai) Co., Ltd., Shanghai, China). Chemiluminescence was measured using an EZ-ECL Chemiluminescence Detection Kit (Sartorius group, Kibbutz Beit-Haemek, Israel) and an ImageQuant LAS-4000 imager (Cytiva, WA, USA). Band intensities were quantified in silico using Aida image analyzer software v4.27 (Raytest, Straubenhardt, Germany). Quantification data were obtained from the results of at least three experiments all showing a similar trend, and typical images are shown.

### 4.9. Statistical Analysis

All experiments described here were repeated three times in a completely randomized design. Statistical calculations were performed using the Statistical Product and Service Solutions program (version 19) (SPSS Inc., Chicago, IL, USA) software. Differences between two datasets were considered significant at the level of *p* < 0.05. The wild type was used as the reference group for all statistical analyses.

## 5. Conclusions

In this study, the yellow-green leaf phenotype of MT is a recessive quality trait. MT showed dramatically decreased photosynthetic pigments content, defective development of chloroplasts, and excessive accumulation of ROS, compared to the wild type. Transcriptomics and metabolomic analyses indicated that most of DEGs and DAMs were involved in photosynthesis, central carbon metabolism, glutathione metabolism, phenylpropanoid synthesis and flavonoid metabolism pathways. The up-regulated expression of chlorophyll (ide) b reductase gene NYC1 and down-regulated expression of four Lhcb genes accelerated the degradation of chlorophyll b and inhibited the LhcB2 protein in MT. Abnormal carbon metabolism and energy metabolism resulted in the significantly declined levels of NADP^+^, D-fructose 6-phosphate, D-glucose 6-phosphate, and citric acid, which may be related to the adaptive enhancement of C_4_ and CAM pathways in MT. In addition, the insufficient supply of NADP^+^, a major acceptor of electrons in PSI, resulted in generation of excessive ROS in MT. The changes in these biological processes may be associated with the yellow-green leaf phenotype of MT. Overall, our findings provide insights into the molecular mechanism underlying the coloration variation in plants.

## Figures and Tables

**Table 1:** Values of malondialdehyde (MDA), hydrogen peroxide (H_2_O_2_), reduced and oxidized glutathione (GSH and GSSH) content, superoxide anion (O_2_^−^) generation rate and superoxidase dismutase (SOD), peroxidase (POD) and catalase (CAT) activity in leaves of mutant (MT) and wild-type plants (WT).

Material	MDA Content (μmol/gFW)	O_2_^−^ Generation Rate (nmol/min·g FW)	H_2_O_2_ Content (μmol/gFW)	POD Activity (U/min·mg Protein)	SOD Activity (U/min·mg Protein)	CAT Activity (U/min·mg Protein)	APX Activity (U/min·mg Protein)	GSH Content (μmol/g FW)	GSSG Content (μmol/g FW)
MT	0.58 ± 0.010 a	127.88 ± 4.059 a	0.744 ± 0.017 a	1.08 ± 0.024 a	14.47 ± 0.103 a	28.58 ± 0.427 a	0.37 ± 0.030 a	49.95 ± 2.183 a	0.09 ± 0.004 b
WT	0.50 ± 0.029 b	26.23 ± 2.622 b	0.565 ± 0.008 b	0.93 ± 0.015 b	13.43 ± 0.222 b	26.98 ± 0.484 b	0.23 ± 0.025 b	41.02 ± 0.350 b	0.129 ± 0.002 a

MT: yellow-green leaf mutant plant of Hami melon; WT: wild-type plant with normal green leaves. The lowercase letters within columns indicate the significant difference at *p* < 0.05.

## Data Availability

The metabolite profiling datasets generated and analyzed during the current study are included in this manuscript and its Supplementary Materials. The transcriptome datasets generated and analysed during the current study have been deposited in the Sequence Read Archive (SRA) at NCBI under the accession number PRJNA784377, [NCBI:PRJNA784377] (https://www.ncbi.nlm.nih.gov/bioproject/?term=PRJNA784377). Requests for material should be made to the corresponding authors.

## Supplementary Materials

The following supporting information can be downloaded at: https://www.mdpi.com/article/10.3390/plants12081623/s1, Table S1: Comparison of agronomic traits between the mutants and wild-type plants, Table S2: Summary of sequencing reads, Table S3: Primer information, Table S4: Description and relative expression of differentially expressed transcription factors in WT vs. MT, Table S5: Protein-Protein Interaction Networks of differentially expressed transcription factors in WT vs. MT (*.ppi.attributes. TXT), Figure S1: GO annotation analysis of DEGs in MT vs. WT, Figure S2: qRT-PCR-based verification of the DEGs in MT vs. WT, Figure S3: KEGG classification statistics of DAMs in MT vs. WT.
