# Supplementary material for: Transcriptomics and Metabolomics Analysis Provides Insight into Leaf Color and Photosynthesis Variation of the Yellow-Green Leaf Mutant of Hami Melon (Cucumis melo L.)
Source: Plants (Basel). 2023 Apr 12;12(8):1623. doi: 10.3390/plants12081623 (PMC10143263; doi:10.3390/plants12081623)
Supplement: Supplementary file 1 [file plants-12-01623-s001.zip › plants-2275828 - Supplementary Table S1-S3 and Figures S1-S3.pdf]

# Supplementary Materials

**Table S1**. Comparison of agronomic traits between the mutants and wild-type plants

| Material | Height (cm) | Stem diameter (mm) | Fresh weight (g) | Dry weight (g) |
|---|---|---|---|---|
| MT | 42.14+9.07b | 9.75+0.34a | 102.79+9.10b | 6.96+0.30b |
| $F_2$ (YGL) | 36.84+4.65b | 9.74+0.22a | 107.70+21.48b | 6.78+1.21b |
| $F_2$ (GL) | 81.26+5.68a | 11.02+1.20a | 371.23+56.99a | 26.64+3.85 a |
| WT | 74.86+11.36a | 10.4 1+1.05a | 345.81+54.09a | 22.88+4.31a |
| F1 | 79.80+14.46a | 10.94+0.57a | 293.17+81.55a | 19.40+5.94a |

Note: Different lowercase letters indicate significant difference at $p<0.05$ level. MT: yellow-green mutant phenotype plants; $F_2$ (YGL): plants with yellow-green leaves in $F_2$ population; $F_2$ (GL): plants with green leaf in $F_2$ population; WT: wild type plants with green leaves.

**Table S2**. Summary of sequencing reads

| sample | Raw reads | Clean reads | Clean bases | Error rate | Q20(%) | Q30(%) | GC(%) |
|---|---|---|---|---|---|---|---|
| WT_1 | 43,403,482 | 42,646,942 | 6.4G | 0.03 | 97.17 | 92.07 | 42.4 |
| WT_2 | 47,531,044 | 46,707,718 | 7.01G | 0.03 | 97.44 | 92.59 | 43.75 |
| WT_3 | 46,970,892 | 45,568,742 | 6.84G | 0.03 | 97.43 | 92.59 | 43.27 |
| MT_1 | 48,891,598 | 47,415,702 | 7.11G | 0.03 | 97.35 | 92.39 | 43.05 |
| MT_2 | 42,645,236 | 41,467,018 | 6.22G | 0.03 | 97.1 | 91.97 | 42.81 |
| MT_3 | 43,136,424 | 42,406,444 | 6.36G | 0.03 | 97.48 | 92.65 | 43.66 |

Note: WT: wild type plants with green leaves; MT: yellow-green mutant phenotype plants.

**Table S3.** Primer information

| Gene name | log2 FC (RNA-Seq) | log2 FC (q-PCR) | Primer information used in the experimental process(F-R) | |
|---|---|---|---|---|
| PCK | 3.203406709 | 5.083333333 | TTGAGAATGTGGTGTTTGATG | CAACGCAGGGGATTTTAG |
| PPDK | 1.590354001 | 2.9 | GAAAAGGAAGGAGTGAGGGT | CAGTTGAGATGGTGAGTCCA |
| PEPC | 1.903984312 | 3.403333333 | GCCACAAGACGAAATGAGA | TGAAAACTGAATAAGAGGAGCA |
| FBP | 1.409818715 | 3.116666667 | TGGAAGGTATTCTGTGGTGTT | TAGGTTAGGTTCGTGGCTGT |
| TIM | 1.283908764 | 2.536666667 | ATCACAGCCCAACCAAAC | GGAAGAGGAGAATCGGAGA |
| MDH | 1.451763137 | 1.1 | TTGCCACCTTGAACCTTT | CTGAACTTTTGAGGGAGTGTT |
| SUS5 | 3.744939345 | 5.883333333 | AAACACACCTTCCAAACTCC | CAATCTCCCCATCCTCTCT |
| TPP | 3.522988072 | 4.433333333 | CACTCTCTGCTTCCGATGA | TGAATCCGTAAACCTTGTCTCT |
| BAM3 | 2.016480218 | 3.653333333 | GTTCCTGTGTTTGTTATGCTTC | GTCCATCTTTCTCCACTAATCC |
| SDHB | 0.955743318 | 2.573333333 | CCGATAGTCCTTCCAAACC | AAGCCAACCCATTACATCC |
| ICDH | 2.27491789 | 4.036666667 | TGTTTTGGTTTGTCCTGATG | TATGCTGTTTGTGCTGGTTT |
| HK1 | 1.635563564 | 2.773333333 | GGAACTGGAACAAATGCTG | CTCGGTGAAGGGAAGATG |
| UGP1 | 1.35962417 | 2.596666667 | TAAAGTGGTTGGCGATGTT | TGAAAGAGTTGTGAAAGGCA |
| CoAOMT | 1.487127562 | 0.873333333 | GGCTATTCGCTCCTCACA | TTACTCCACCAACCTTCACC |
| CYP98A2 | 0.870067651 | 1.206666667 | CAAACTCTACCACCGTCTCC | ACCTCCCTCGCTAACTCC |
| GSHPx | 1.344567314 | 3.196666667 | CCTGCTCAAGGTTCAAGG | AAAAGGCGATGTTGTTGG |
| ODC1 | 1.713576754 | 1.383333333 | CCTTACCACTTTTGATTCCG | GCCGTATTTGTTTCCCAA |
| PWD | 1.127128268 | 2.52 | TACACACATACAGCCCAACC | ACTACACCATCAAACTTCCCA |

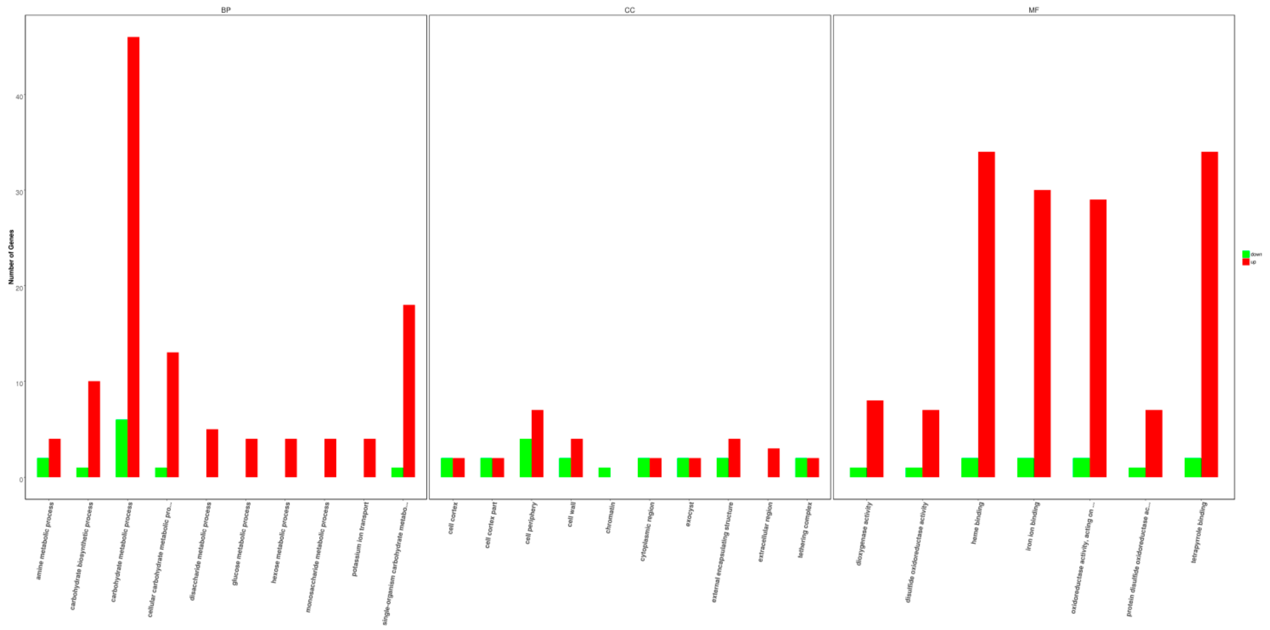


**Figure S1.** GO annotation analysis of DEGs in MT vs. WT. BP, biological processes; CC, cellular components; MF, molecular functions. MT: yellow-green mutant phenotype plants; WT: wild type plants with green leaves.

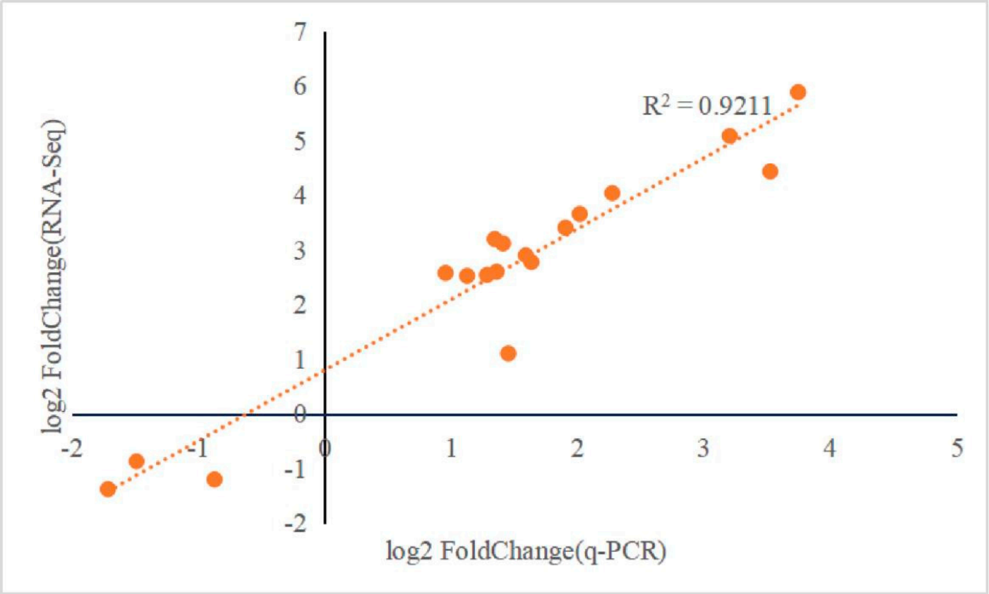


**Figure S2.** qRT-PCR-based verification of the DEGs in MT vs. WT. MT: yellow-green mutant phenotype plants; WT: wild type plants with green leaves.

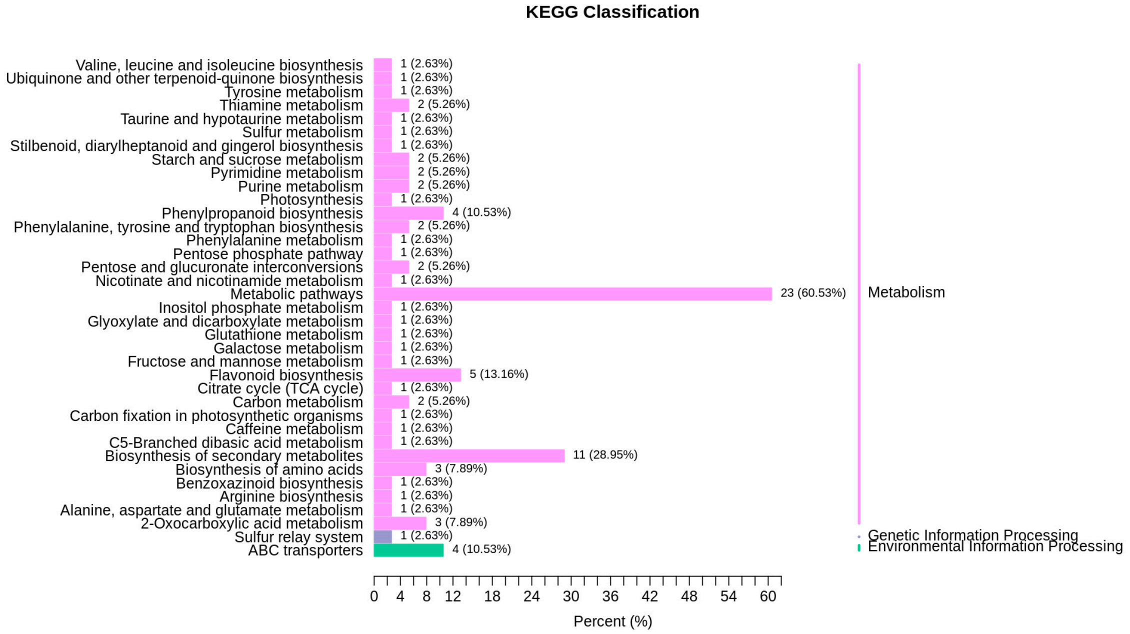


**Figure S3.** KEGG classification statistics of DAMs in MT vs. WT. MT: yellow-green mutant phenotype plants; WT: wild type plants with green leaves.
